# Can Cranberry Juice Protect against Rotenone-Induced Toxicity in Rats?

**DOI:** 10.3390/nu13041050

**Published:** 2021-03-24

**Authors:** Monika Kurpik, Przemysław Zalewski, Małgorzata Kujawska, Małgorzata Ewertowska, Ewa Ignatowicz, Judyta Cielecka-Piontek, Jadwiga Jodynis-Liebert

**Affiliations:** 1 Department of Toxicology, Poznan University of Medical Sciences, Dojazd 30, 60-631 Poznań, Poland; m.kurpik@wp.pl (M.K.); mewertow@ump.edu.pl (M.E.); liebert@ump.edu.pl (J.J.-L.); 2 Department of Pharmacognosy, Poznan University of Medical Sciences, Święcickiego 4, 60-781 Poznań, Poland; pzalewski@ump.edu.pl (P.Z.); jpiontek@ump.edu.pl (J.C.-P.); 3 Department of Pharmaceutical Biochemistry, Poznan University of Medical Sciences, ul. Święcickiego 4, 60-781 Poznań, Poland; eignato@ump.edu.pl

**Keywords:** oxidative stress, liver, kidney, brain, serum, polyphenols, HPLC analysis

## Abstract

The high polyphenols content of cranberry accounts for its strong antioxidant activity underlying the beneficial health effects of this fruit. Rotenone (ROT) is a specific inhibitor of mitochondrial complex I in the brain which leads to the generation of oxidative stress. To date, there are few data indicating that toxicity of ROT is not limited to the brain but can also affect other tissues. We aimed to examine whether ROT-induced oxidative stress could be counteracted by cranberry juice not only in the brain but also in the liver and kidney. Wistar rats were given the combined treatment with ROT and cranberry juice (CJ) for 35 days. Parameters of antioxidant status were determined in the organs. ROT enhanced lipid peroxidation solely in the brain. The increase in the DNA damage was noticed in all organs examined and in leukocytes. The beneficial effect of CJ on these parameters appeared only in the brain. Additionally, CJ decreased the activity of serum hepatic enzymes. The effect of CJ on antioxidant enzymes was not consistent, however, in some organs, CJ reversed changes evoked by ROT. Summing up, ROT can cause oxidative damage not only in the brain but also in other organs. CJ demonstrated a protective effect against ROT-induced toxicity.

## 1. Introduction

Cranberry fruit contains high quantities of polyphenols such as anthocyanins, flavonols, and proanthocyanidins. All these compounds possess strong antioxidant properties, which have been demonstrated mainly in in vitro experiments, and it is suggested that beneficial health effects of cranberry are ascribed, to a large extent, to this activity [1].

The most commonly studied and best-recognized cranberry health effect is its antibacterial capability in protecting urinary tract infections [1,2]. Proanthocyanidin A, a unique compound not found in other fruit, is responsible for inhibiting pathogenic bacteria adhesion to uroepithelial cells [3].

The antioxidant potency of cranberry has been demonstrated in experiments on animal models in which oxidative stress has been generated. Oxidative stress evoked in rats fed the atherogenic diet and injected with lipopolysaccharide was partially attenuated by the administration of cranberry powder in the diet for six weeks [4]. Cranberry protected against doxorubicin-induced cardiotoxicity in rats by inhibiting the depletion of cardiac glutathione and an increase in malondialdehyde and protein carbonyls levels [5]. Administration of cranberry extract resulted in beneficial changes of serum oxidative stress parameters in rats treated with model pro-oxidant, carbon tetrachloride [6]. Oxidative stress-induced in rats by a high-fat cholesterol diet [7] and obesity [8] was attenuated by several weeks of consumption cranberry preparations.

Clinical studies have demonstrated that cranberry products can protect against some chronic disorders by reducing cardiovascular risk factors, improving glucoregulation and endothelial function, reducing biomarkers of metabolic syndrome and downregulating inflammatory biomarkers [2,9,10,11].

Additionally, cranberry preparations have been shown to inhibit the growth of several types of cancer cell lines [12,13,14]. A single report referring to anticancer properties of cranberry in the animal model has been found in the available literature. In rats challenged with the carcinogen N-butyl-N-(4-hydroxybutyl)-nitrosamine and treated chronically with cranberry juice, substantial reduction of urinary bladder cancer number has been observed [15].

Rotenone (ROT), a lipophilic natural compound, is a specific inhibitor of mitochondrial complex I and impairs the mitochondrial electron transport system, leading to ROS’s bulk production. The contribution of oxidative stress to rotenone toxicity has been evidenced in numerous in vitro and in vivo experiments [16]. ROT is commonly used as a model neurotoxin since it causes in rodents symptoms resembling Parkinson’s disease (PD) in humans [17,18]. For a long time, research interest has been focused on neurotoxic properties of ROT. However, recently the toxicity of ROT towards other tissues/organs has been demonstrated. Ravenstijn et al. (2008) reported significant histopathological changes in the stomach and minor in rats’ liver and heart treated with ROT [19]. Significant pathology was also observed in the liver, kidney, lung, and spleen of rats administered ROT [20]. These findings have been confirmed by Zhang et al. (2017), who reported that ROT caused similar pathologic changes in the mentioned above organs of the rat [21]. The pro-oxidant action of ROT in the liver of rats has been manifested in an increased level of malondialdehyde (MDA) and a decreased activity of two antioxidant enzymes, superoxide dismutase and catalase [22]. Abdel-Salam et al. (2017) demonstrated marked vacuolar degeneration in hepatocytes as well as edema and atrophy of epithelial lining of tubules in the kidney of ROT-treated rats [23]. Since mitochondrial dysfunction contributes to the development of chemical toxicity and disease processes, ROT has also been applied in animal models of chemically induced toxicity for evaluation of the protective effects of plant extracts [23,24] and the explanation of the toxicity mechanisms of drugs [25,26]. Moreover, ROT is still used as a pesticide in some regions of the world [24]; hence environmental exposure might also be considered.

Given cited above findings, we aimed to investigate whether oxidative stress evoked by ROT could be counteracted by cranberry juice not only in the brain but also in two other essential organs, the liver, and kidney. We examined oxidative stress parameters in the organs as well as serum markers of liver function.

## 2. Materials and Methods

Commercial 6-fold concentrated cranberry juice (CJ) was obtained from Alter Medica (Zywiec, Poland). The product was manufactured in accordance with the principle of HACCP (hazard analysis and critical control point) and fruit ingredients are fully compliant with the Code of Practice of the European Fruit Juice Association (AIJN).

### 2.1. HPLC Analysis of Cranberry Juice (CJ)

Analysis was performed on (Thermo Scientific UltiMate 3000 UHPLC) system equipped with UV detector. Zorbax Eclipse Plus C18 column (100 × 4.6 mm, 3.5 μm) was used. The mobile phase consisted of acetonitrile (A) and 0.1% formic acid (B). The linear gradient profile was as follows: 10–20% (A) for 45 min, 20–30% (A) for 15 min, 30–40% (A) for 10 min, and 40–10% for 10 min. The flow rate of mobile phase was set at 0.8 mL/min, the temperature was 40 °C. The detection wavelength was 240, 254, 270, and 520 nm. The identification of each compound was carried out by the comparison of both retention time and UV-VIS spectra of reference compound. Epicatechin and cyanidin-3-O-glucoside, analytical standards were obtained from ChromaDex. Quercetin (purity ≥ 95%) and chlorogenic acid (purity 96.6%) were purchased from Sigma-Aldrich and Dr. Ehrenstorfer GmbH respectively.

### 2.2. Animals

The animal experiment was performed on six-week-old male albino Wistar rats weighing 250–300 g. All the animals used in this study were bred in the Department of Toxicology, Poznan University of Medical Sciences (Poznań, Poland). Animals were held (four rats/cage) in polycarbonate cages (Tecniplast, Buguggiate, VA, Italy) with wood shavings in a room maintained under 12 h light/dark cycle, 22 ± 2 °C, 40–54% relative humidity, and controlled circulation of air. A commercial diet (ISO 22000 certified laboratory feed Labofeed H) and drinking water were available ad libitum.

### 2.3. Experimental Design

In order to induce PD in rats, rotenone (ROT, Sigma-Aldrich, Poznań, Poland) was injected subcutaneously once daily for 35 days in a dose of 1.3 mg/kg body weight [27]. Fifty rats were divided randomly into five groups, with 10 animals in each. Group I: rats receiving water (i.g.) and helianthi oleum raffinatum (FAGRON a.s., Olomouc, Czech Republic) (s.c.) from the 11th day, designated as a control group (Control). Group II: rats treated with cranberry juice alone in a dose of 500 mg/kg b.w./day (i.g.), designated as cranberry juice-treated group (CJ II). Group III: rats injected with rotenone in helianthi oleum raffinatum (1.3 mg/kg b.w./day, s.c.) alone from the 11th day of the experiment, designated as rotenone group (ROT). Groups IV and V: rats treated with cranberry juice in a dose of 200 and 500 mg/kg b.w./day (i.g.), respectively, and injected with rotenone from the 11th day, designated as cranberry juice + rotenone group (CJ I + ROT, CJ II + ROT). The experiment lasted a total of 45 days, including 10 days pre-treatment with CJ and 35 days combined treatment with CJ and ROT. The animals were observed daily for clinical signs of toxicity, and body weight was recorded weekly. Twenty-four hours after the last treatment, the rats were anesthetized with ketamine/xylazine (100 U/7.5 mg/kg b.w., intraperitoneally), blood was withdrawn from the heart. Following intracardiac perfusion with isotonic sodium chloride solution, the brain, liver, and kidney were removed quickly and stored at −80 °C until further use.

### 2.4. Biochemical Examinations

#### 2.4.1. Sample Collection

A portion of whole heparinized blood was separated for the comet assay; the remaining blood was centrifuged at 2000× *g* for 10 min in a refrigerated centrifuge.

Frozen tissues were homogenized with a lysis buffer (Cell Lysis Buffer 2; Bio-Techne-R&D Systems, Minneapolis, MN, USA) supplemented with a cocktail of protease and phosphatase inhibitor (Protease Inhibitor Cocktail I, Bio-Techne-Tocris, Minneapolis, MN, USA) at a weight:volume ratio of 1:2, using a tissue homogenizer (Ultra-Turrax model T25; Ika Labortechnik, Staufen, Germany). The homogenate of each sample was centrifuged at 10,000× *g* for 20 min at 4 °C. The supernatant was collected for the biochemical assays with the exception of mitochondrial ALDH2 activity assay.

A portion of whole heparinized blood was centrifuged (3000 rpm, 4 °C) for plasma separation.

#### 2.4.2. Oxidative Stress Markers

Lipid peroxidation was determined by the reaction of its end product MDA with thiobarbituric acid (TBA) according to the manufacturer’s protocol provided with the lipid peroxidation (MDA) assay kit (Sigma-Aldrich, Poznań, Poland).

An alkaline comet assay was conducted according to the method of Hartmann et al. [28] as previously described [29].

The reduced glutathione (GSH) level and antioxidant enzyme activities were determined spectrophotometrically as described by Kujawska et al. [29,30]. Briefly, GSH was quantified with Ellman’s reagent. The superoxide dismutase (SOD) activity was measured using spontaneous epinephrine oxidation. The catalase (CAT) activity was assayed by the measurement of hydrogen peroxide reduction–oxidation. Glutathione peroxidase (GPx) and glutathione reductase (GR) activities were determined by measuring NADPH oxidation using hydrogen peroxide as a substrate and in the presence of oxidized glutathione, respectively. Glutathione S-transferase (GST) activity was determined using 1-chloro-2,4-dinitrobenzene (CDNB) as a substrate. Paraoxonase-1 (PON-1) activity was measured with phenylacetate as a substrate; the rate of phenol generation was a measure of the enzyme activity. The activity of heme oxygenase-1 (HO-1) and NAD(P)H quinone oxidoreductase 1 (NQO1) were assayed according to the published methods based on the reduction of biliverdin into bilirubin [31] and by using cytochrome c as an electron acceptor in the presence of NADPH [32], respectively.

Total antioxidant status (TAS) was measured as trolox equivalent antioxidant capacity (TEAC), with a modification of the ABTS-cation-radical method [33].

#### 2.4.3. Mitochondrial Aldehyde Dehydrogenase (ALDH2) Activity

The activity of this enzyme was determined using the ALDH2 activity assay kit according to the manufacturer’s protocol (ab115348, Abcam). Tissue samples of the brain were homogenized in three-volumes of ice-cold phosphate-buffered saline. An extraction buffer was added to the homogenate to get protein concentration of 10 mg/mL and centrifuged at 16,000× *g* 4 °C for 20 min followed by incubation on ice for 20 min. The samples in the volume of 100 μL were then subjected to a procedure of microplate assay.

#### 2.4.4. Protein Determination

The quantity of protein in samples was measured employing the Bicinchoninic Acid Protein Assay Kit following the manufacturer’s instruction (BCA1 AND B9643, Sigma-Aldrich, Poznań, Poland).

#### 2.4.5. Liver Function Markers

Hepatic enzyme activity (alanine aminotransferase, ALT; aspartate aminotransferase, AST; alkaline phosphatase, ALP, and lactate dehydrogenase, SDH) were determined in plasma according to the reagent kit’s manufacturer instructions (Pointe Scientific, Warszawa, Poland).

## 3. Results

### 3.1. Determination of Polyphenols in Cranberry Juice (CJ)

**Figure 1:**
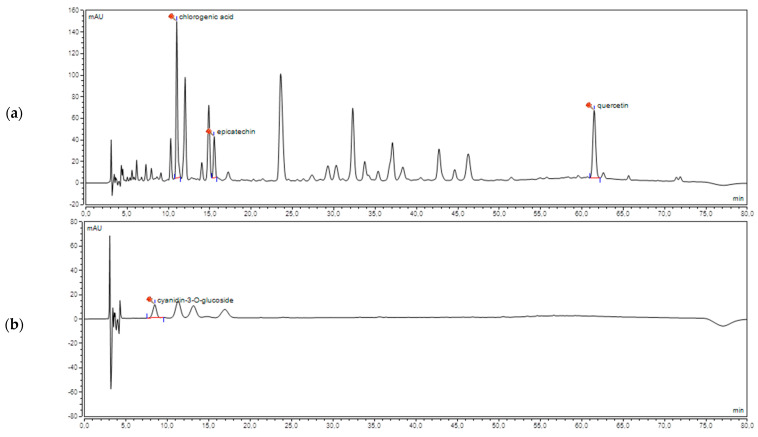
The chromatogram of cranberry juice (CJ): detection wavelength 270 (**a**) and 520 (**b**).

Due to the analysis of a complex and difficult plant matrix different mobile and stationary phases were examined to obtain the best separations of the cranberry juice components. Analysis on LiChrospher 100 RP-18 (250 × 4, 5 μm) column yielded too long retention time, whereas the use of Kinetex C18 column (100 × 2.1 mm, 5 μm) shortened this parameter but the best separations were obtained using Zorbax Eclipse Plus C18 column (100 × 4.6 mm, 3.5 μm). Subsequently, the influence of various concentrations of acetonitrile, methanol, ammonium acetate, water, and formic acid on retention time and selectivity were analyzed. The best separation was obtained with gradient elution (acetonitrile and 0.1% formic acid). Due to the prolonged retention time and peak broadening methanol was eliminated as a component of the mobile phase in further research. The change of formic acid to water or ammonium acetate in the mobile phase composition did not significantly affect the separation parameters. As a result of the optimization steps, the components of cranberry juice were separated for about 70 min. The method was selective for the following markers: quercetin, epicatechin, chlorogenic acid, cyanidin-3-O-glucoside (Figure 1). Their concentrations were 15.69, 10.02, 3.90, 0.37 mg/L respectively.

### 3.2. Biochemical Measurements

#### 3.2.1. Effects of Cranberry Juice on the Oxidative Stress Parameters in the Brain of Rats Treated with Rotenone

Malondialdehyde (MDA) concentration was increased by 86% in ROT-treated rats as compared to controls. Both doses of CJ caused a decrease in MDA level by 79% and 75%, respectively, compared to the ROT- challenged group. Four enzymes were also affected by ROT: the activity of GST was increased by 44%, SOD activity by 43%, GR activity by 55%, and mitochondrial aldehyde dehydrogenase activity was decreased by 21% as compared to controls. Combined treatment with ROT and CJ decreased the activity of GR by 65% and 71%; other enzymes were not affected in these groups. Although CAT activity was not changed by ROT, combined treatment resulted in an 82% increase in the enzyme activity compared to controls (Table 1).

Treatment with CJ alone resulted in changes in three parameters: a 66% decrease in MDA level, a 72% increase in SOD activity, and a 76% increase in GR activity. Comet assay revealed that ROT enhanced the basal level of DNA damage by 54%. Both doses of cranberry juice attenuated this damage by 19% and 28%, respectively (Table 1).

GPx activity and the content of GSH were affected neither by ROT nor by combined treatment (data not shown).

#### 3.2.2. Effects of Cranberry Juice on the Oxidative Stress Parameters in the Liver of Rats Treated with Rotenone

The response of antioxidant enzymes to rotenone in the liver was diversified. Rotenone did not affect SOD and CAT; however, their activities were enhanced in the groups receiving combined treatment by 25–85% compared to the ROT group. GPx, HO-1, and NQO1 activities were increased by 73%, 80%, and 141%, respectively, in the ROT group and remained at a similar level in the groups receiving the combined treatment. The GR activity changes had an opposite direction: ROT caused the slight, 22%, decrease which remained at the same level (about 20%) in the groups undergoing combined treatment. PON 1 activity was changed exclusively in rats treated simultaneously with cranberry juice and ROT—low dose of CJ caused a 35% decrease, whereas, in the high-dose group, an 82% increase was observed compared to the ROT-treated group (Table 2).

Several enzyme activities were increased by cranberry juice alone: SOD by 70%, GPx by 59%, and NQO1 by 100%, as compared to controls. The range of DNA damage in the ROT group vs. control was increased by 68% and remained unaffected by simultaneous treatment with cranberry juice (Table 2). The concentration of MDA, GSH, and GST activity in all experimental groups did not differ from controls (data not shown).

To examine the effect of ROT and CJ on liver function, hepatic enzyme activities in serum were determined. The results were very consistent and reflected liver function impairment by ROT and the beneficial action of cranberry juice. The activity of all enzymes: ALT, AST, ALP, and LDH was increased by 31–220% in rotenone-treated rats. In groups receiving combined treatment, the activity of ALT, AST, and LDH was decreased by 30–51% compared to the ROT group; however, it did not return to the values noted in the controls (Table 3).

#### 3.2.3. Effects of Cranberry Juice on the Oxidative Stress Parameters in the Kidneys of Rats Treated with Rotenone

The pattern of the antioxidant enzymes activities in the kidney of rotenone-treated rats differs from that in other organs examined. The activity of GPx and GR was increased by 38% and 11%, respectively, as compared to controls, whereas the CAT and PON1 activities were decreased by 31% and 15%, respectively. Simultaneous administration of both CJ doses reversed these changes, with the exception of GPx, for which activity did not differ statistically from that in the ROT group, even to the control level for PON1 activity (Table 4).

GSH concentration increased by 80% in ROT-treated rats and was slightly (by 17%) reduced in the group receiving ROT and the higher CJ dose. Cranberry juice alone did not affect any of the parameters tested (Table 4).

The range of DNA damage in the ROT group increased by 91% vs. control, and co-treatment with the juice did not significantly change this value. MDA level and SOD, GST, HO-1, NQO1 activities were not changed in any experimental groups (data not shown).

#### 3.2.4. Effects of Cranberry Juice on the Oxidative Stress Parameters in the Serum of Rats Treated with Rotenone

Very few parameters in serum were affected by the treatment. ROT caused a 27% decrease in the value of total antioxidant status, which was enhanced/improved slightly (15%) by the higher CJ dose. DNA damage in leukocytes was substantially increased (78%) by ROT administration and was not reversed by simultaneous treatment with both CJ doses. Some enzymes activity was changed only in groups undergoing combined treatment. GPx activity was significantly reduced by both doses of CJ by 35% and 91%, respectively, as compared to controls and the ROT-treated group. Conversely, PON1 activity in these groups was about 20% higher vs. controls and ROT-treated group. The decrease in SOD activity (17%) was noted in the CJII + ROT group (Table 5).

There were no differences in the CAT, GR, and GST activities between controls and treated groups (data not shown).

## 4. Discussion

### 4.1. Brain

As could be expected from the ROT mechanism of action, lipid peroxidation in the brain measured as MDA concentration was enhanced in ROT-treated rats. In other authors’ reports, the same effect has been observed in rats regardless of the dose used (0.5–3 mg/kg), the route of administration (ip or sc), or the period of exposure (7–35 days) [34,35,36,37,38,39,40,41,42,43,44]. The antioxidant action of cranberry juice resulted in a distinct decrease in MDA concentration, yielding the values noticed in the controls.

GSH content in the brain was not changed in any experimental group, conversely to cited above reports in which GSH depletion has been observed in ROT treated rats. However, the opposite response of GSH in the brain, namely, an increase in its content, has been reported in mice [45] or rats [46] treated with a similar dose of ROT for 21 and 11 days, respectively.

The protective effect of cranberry juice against ROT induced oxidative DNA damage was evidenced by the dose-dependent decrease in this parameter in rats undergoing combined treatment.

The observed in the present study response of antioxidant enzymes to ROT was diversified, similarly as in other reports. In the vast majority of publications concerning the effect of ROT on antioxidant enzymes, their activities have been reduced [34,35,36,37,38,39,40,42,43,44,47,48,49,50]. However, several reports can be found in the available literature where ROT has evoked increased activities of several enzymes [41,45,46,51] or caused no alterations in their activities [37,49,51,52].

ROT has been found to induce the activation of the transcription factor Nrf2 pathway leading to the enhanced expression of genes that regulate antioxidant defense mechanisms, including antioxidant enzymes [53]. Hence, the increase in the activity of some antioxidant enzymes demonstrated in the present study, and other reports might be interpreted as an adaptive response of the brain tissue to oxidative insult [46]. However, it remains unclear why, in similar experiments, these enzymes’ activity has been suppressed.

Given the specific role of aldehyde dehydrogenase 2 (ALDH2) in the brain, the determination of this enzyme was included in the set of parameters examined in the brain tissue. Mitochondrial ALDH2 catalyzes detoxification of biogenic aldehydes such as malondialdehyde and 4-hydroxynonenal derived from lipid peroxidation. Additionally, ALDH2 is involved in the metabolism of catecholamines: dopamine (DA), norepinephrine, and epinephrine. In the brain, DA is oxidized to 3,4-dihydroxyphenylacetaldehyde (DOPAL), a very reactive and toxic molecule, leading to enhanced ROS formation and mitochondrial damage in dopaminergic neurons, which contributes to the pathogenesis of Parkinson disease. ALDH2 detoxifies DOPAL to form non-active 3,4-dihydroxyphenylacetic acid [54]. Goldstein et al. (2015) have found that rotenone attenuates ALDH2 activity in nerve cells in vitro, explaining some aspects of rotenone neurotoxicity. The present study results support their findings since a decrease in the ALDH2 activity in the ROT treated rats was noticed [55].

Cranberry juice did not substantially affect changes in antioxidant enzymes activity caused by ROT, with the exception of GR. The enzyme activity was distinctly decreased in rats receiving combined treatment and reached the values lower than that noticed in the controls. This effect might be explained by the concept of “adaptive homeostasis,” assuming that the moderate oxidative stress caused activation of GR, the enzyme involved in maintaining GSH in its reduced form. The attenuation of oxidative stress by the juice tested resulted in decreased enzyme activity [56]. On the other hand, it is probable that some interactions between ROT and CJ occurred since both substances, when given alone, increased the GR activity, but their combination caused an opposite effect.

### 4.2. Liver

Since ROT is considered to be a specific neurotoxin, its organ toxicity has not been widely investigated. Ravenstijn et al. (2008) have reported histopathological changes in the stomach and liver of rats treated subcutaneously with ROT (3 mg/kg/day) for 14 and 28 days [19]. Peripheral organ pathology has also been revealed in rats treated with ROT (2 mg/kg/day, s.c.) for five weeks [20]. The authors have observed changes in the kidney, lung, spleen, and the most distinct in the liver: neutrophil infiltration in the periportal areas, the expansion of central veins and bile ducts. Similar effects have been described by Zhang et al. (2017) in rats undergoing the same treatment with ROT; fatty degeneration of hepatocytes, neutrophilic infiltration in the pulmonary alveoli, and white pulp hyperplasia in the spleen [21].

The present study supports, to some extent, these findings, since all determined hepatic serum enzymes were increased considerably in ROT treated rats indicating the impairment of the liver function. Surprisingly, lipid peroxidation level and GSH content were not affected by ROT, whereas an increase in DNA damage proved that oxidative insult occurred in the organ.

Besides commonly determined antioxidant enzymes, we included in the set of parameters assayed additional three enzymes abundantly expressed in the liver. Heme oxygenase-1 (HO-1) catalyzes heme degradation resulting in the formation of iron, carbon monoxide, and biliverdin. HO-1 activity is very high in the liver. Additionally, the liver is the second most active heme-producing tissue [57]. The antioxidant, anti-inflammatory, and cytoprotective functions of HO-1 are attributable to its degradation products. HO-1 is a highly inducible enzyme by heme and other stimuli, including oxidative stress [58], and confers protection against oxidative tissue injuries [59]. It is thought that HO-1 activation appears to be a general indicator of oxidative stress in cells [60].

NAD(P)H: quinone reductase 1 (NQO1) is very effective in catalyzing detoxification of quinones by the two-electron mediated reduction to hydroquinones, thus preventing quinones from redox cycling. The enzyme is also a component of the plasma membrane redox system generating antioxidant forms of ubiquinone and vitamin E. NQO1 is an inducible protein and can increase considerably under stress conditions as a member of the adaptive cellular response [61]. Transcription of HO-1 and NQO1, similarly to glutathione-related antioxidant enzymes, is regulated by the transcription factor Nrf2 and is enhanced in response to stress [62].

Paraoxonase 1 (PON1) is synthesized by the liver and released into the serum, where it is associated with HDL lipoproteins. PON1 is a hydrolytic enzyme with a wide range of substrates and the capability to protect against lipid peroxidation and decompose hydrogen peroxide and lipid peroxides [63]. The enzyme is primarily expressed in the liver; however, it is also localized in other tissues. Some bioactive molecules such as dietary polyphenols are known to stimulate PON1 transcription in mouse liver and HepG2 cell line [63].

The response of antioxidant enzymes to ROT was diversified. Only activities of enzymes which transcription is regulated by Nrf2, namely GPx, HO-1, and NQO1, were substantially increased, suggesting a compensatory response to protect the cell from damage induced by ROT pro-oxidative action [51]. This effect was consistent with the observed increased level of DNA damage. It could be suggested that oxidative insult exerted by ROT in the liver was of weak magnitude and resulted in enhanced activities of the inducible enzymes and did not cause oxidative damage to other enzymes except for a slight decrease in GR activity.

A single publication referring to ROT-induced changes in hepatic antioxidant status has been found in the available literature [22]. The authors have observed an increased MDA level and diminished activities of SOD and CAT in the liver of rats treated with ROT (1 mg/kg/day, i.p.) for 60 days. Our results did not support these findings what is probably due to differences in experimental design.

The effects of the juice alone cannot be overlooked. Activities of several enzymes were induced by polyphenols present in the juice, enhancing the antioxidant status in the liver.

Combined treatment yielded increased activities of SOD, CAT, and PON 1. CAT activity was affected neither by juice alone nor by ROT, which suggests that some interaction occurs, resulting in enhancing activity in rats undergoing combined treatment.

### 4.3. Kidneys

Admittedly the MDA content was not increased in the kidneys of rats treated with ROT; however, the increase in DNA damage indicated that antioxidant status was impaired. A considerable increase in the content of GSH corroborates this finding. It could be considered a compensatory response to a decrease in the level of cellular GSH that probably occurred in an earlier phase of the experiment [30]. Treatment with a higher dose of juice ameliorated this overcompensation, which can be interpreted as returning to the redox balance.

The response of antioxidant enzymes to ROT was different than in the liver except for GPx, whose activity was enhanced in both organs. The magnitude of alterations was relatively small: a decrease in the CAT and PON1 activities and an increase in GR activity. No changes in HO-1 and NQO1 activities were noticed in comparison with the controls. As discussed above, both enzymes respond to oxidative stress increasing their activity. Presumably, kidneys were less affected by ROT than the liver. On the other hand, the effect of the juice tested on CAT, GR, and PON1 was very consistent—it reversed changes evoked by ROT yielding values similar to those in controls. It could be interpreted as an improvement of antioxidant status regardless of the direction of changes.

### 4.4. Serum

Plasma TAC determined by the ABTS assay is considered to be an appropriate method to measure overall plasma antioxidant capacity and predict the body’s antioxidant status [64]. Deterioration of the antioxidant status of rats treated with ROT was evidenced in the present study by the decrease in plasma TAC value and an increased rate of DNA damage in lymphocytes. The juice treatment partially improved only the former parameter.

Antioxidant enzyme activities in plasma are commonly determined in animal experiments to examine the induction of oxidative stress by toxicants or diseases and protective effects of natural compounds of plant origin [65,66,67,68,69,70]. Conversely to the findings in tissues, ROT did not affect any of the enzymes assayed what could be ascribed to the low level of oxidative stress.

Cranberry juice affected the activity of SOD, GPx, and PON1 only in the groups of rats receiving combined treatment; however, the effects were equivocal. Whereas the increase in PON1 activity can be considered beneficial, a significant decrease in GPx activity attenuated the antioxidant defense system.

## 5. Summary and Conclusions

As mentioned above, ROT inhibits mitochondrial complex I what results in the production of ROS. The present study demonstrated the protective effects of cranberry juice against oxidative damage exerted by ROT to lipids and DNA in the brain. Our preliminary studies support these findings since we have demonstrated that cranberry juice significantly decreased the degeneration of neurons in the midbrain of rotenone-treated rats (unpublished data). Data concerning antioxidant enzymes appeared inconclusive since the majority of their activities in the brain were not affected by the juice.

Our study revealed that exposure to ROT evoked DNA damage and brought about the diversified response of antioxidant enzymes not only in the target organ but also in the liver, kidney, and leukocytes. Additionally, the increased activities of the serum hepatic enzymes were observed, indicating the impairment of liver function. These results corroborate earlier findings concerning pathologic changes in several organs of rodents treated with ROT. The protective effect of cranberry juice was evidenced by the decrease in serum hepatic enzymes as well as by normalization of some antioxidant enzyme activities.

Surprisingly, rotenone also affected blood leading to DNA damage in leukocytes and a decreased value of total antioxidant status in serum. The juice treatment partially protected only the latter parameter.

Our results confirm the commonly accepted view that moderate levels of ROS evoke rises in antioxidant enzymes considered to be a kind of adaptive response while high levels of oxidative stress reduce enzyme activities as a result of damage to the molecular system involved in the induction of these enzymes [71,72]. Since sex is considered a biological variable in preclinical research, it should be emphasized that our findings are limited to male rats.

Among all results presented in our work, several examples show that enzyme activities were altered solely in rats undergoing the combined treatment. It can be suggested that some interactions occurred between rotenone and the components of juice concerning either activity modulation or expression level.

## Figures and Tables

**Table 1:** Effect of cranberry juice (CJ) on oxidative stress markers in the brain of rotenone (ROT) injected rats.

Parameters	Treatment				
	Control	CJ II	ROT	CJ I + ROT	CJ II + ROT
SOD [U/mg protein]	39.40 ± 4.37	67.62 ± 16.48 ^(a)^	56.17 ± 10.91 ^(a)^	52.31 ± 12.38	66.45 ± 16.19
CAT [U/mg protein]	0.60 ± 0.23	0.85 ± 0.27	0.77 ± 0.23	1.06 ± 0.26	1.09 ± 0.24
GR [nmol NADPH/min/mg protein]	0.33 ± 0.09	0.58 ± 0.13 ^(a)^	0.51 ± 0.11 ^(a)^	0.18 ± 0.04 ^(b)^	0.15 ± 0.03 ^(b)^
GST [nmol CDNB/min/mg protein	362.4 ± 96.6	480.8 ± 110.3	523.4 ± 128.0 ^(a)^	577.8 ± 170.9	495.2 ± 92.2
ALDH2 [nmol NADPH/min/mg protein]	7.95 ± 0.65	6.93 ± 0.98	6.31 ± 0.43 ^(a)^	6.52 ± 1.12	7.11 ± 0.93
MDA [nmol/g tissue]	48.3 ± 8.7	16.5 ± 1.9 ^(a)^	89.65 ± 55.0 ^(a)^	18.8 ± 6.6a ^(b)^	22.3 ± 4.9 ^(b)^
DNA damage [arbitrary points]	112 ± 6	111 ± 3	172 ± 10 ^(a)^	140 ± 4 ^(b)^	124 ± 4 ^(b)^

^(a)^—*p* < 0.05 vs. Control. ^(b)^—*p* < 0.05 vs. ROT.

**Table 2:** Effect of cranberry juice (CJ) on oxidative stress markers in the liver of rotenone (ROT)-injected rats.

Parameters	Treatment				
	Control	CJ II	ROT	CJ I + TOT	CJ II + ROT
SOD [U/mg protein]	27.7 ± 5.0	47.1 ± 12.4 ^(a)^	32.8 ± 11.0	52.8 ± 7.8 ^(b)^	60.8 ± 9.7 ^(b)^
CAT [U/mg protein]	2.55 ± 0.37	3.02 ± 0.67	2.83 ± 0.57	3.97 ± 0.73 ^(b)^	3.55 ± 0.50
GPx [nmol NADPH/min/mg protein]	285.7 ± 62.6	453.0 ± 90.7 ^(a)^	493.8 ± 95.2 ^(a)^	485.8 ± 71.8	502.9 ± 79.0
GR [nmol NADPH/min/mg protein]	72.1 ± 8.8	69.9 ± 5.9	56.4 ± 9.9 ^(a)^	54.3 ± 7.3	56.7 ± 6.7
HO-1 [pmol bilirubin/min/mg protein]	13.2 ± 3.2	17.9 ± 5.5	23.7 ± 7.2 ^(a)^	21.7 ± 8.4	20.0 ± 6.7
NQO1 [nmol cyt c/min/mg protein]	8.8 ± 2.9	17.6 ± 3.2 ^(a)^	21.2 ± 3.5 ^(a)^	23.4 ± 6.6	21.3 ± 6.2
PON1 [μmol phenol/min/mg protein]	722.5 ± 92.6	814.1 ± 92.3	807.3 ± 66.3	526.3 ± 39.1 ^(b)^	1470.9 ± 120.3 ^(b)^
DNA damage [arbitrary points]	73 ± 3	78 ± 3	123 ± 4 ^(a)^	118 ± 5	121 ± 4

^(a)^—*p* < 0.05 vs. Control. ^(b)^—*p* < 0.05 vs. ROT.

**Table 3:** Effect of cranberry juice (CJ) on liver function markers in rotenone (ROT)-injected rats.

Parameters	Treatment				
	Control	CJ II	ROT	CJ I + ROT	CJ II + ROT
ALT [U/L]	20.6 ± 1.8	22.7 ± 1.7	39.3 ± 5.5 ^(a)^	39.6 ± 3.9	27.6 ± 3.6 ^(b)^
AST [U/L]	82.6 ± 10.7	84.3 ± 12.6	236.7 ± 28.3 ^(a)^	97.6 ± 8.5 ^(b)^	115.6 ± 11.5 ^(b)^
ALP [U/L]	78.7 ± 9.2	104.3 ± 10.2 ^(a)^	102.9 ± 15.1 ^(a)^	114.6 ± 15.8	111.1 ± 17.9
LDH [U/L]	128.9 ± 16.7	138.2 ± 16.4	412.9 ± 54.5 ^(a)^	247.0 ± 38.0 ^(b)^	185.7 ± 29.1 ^(b)^

^(a)^—*p* < 0.05 vs. Control. ^(b)^—*p* < 0.05 vs. ROT.

**Table 4:** Effect of cranberry juice (CJ) on oxidative stress markers in the kidneys of rotenone (ROT)-injected rats.

Parameters	Treatment				
	Control	CJ II	ROT	CJ I + TOT	CJ II + ROT
CAT [U/mg protein]	0.16 ± 0.01	0.17 ± 0.01	0.11 ± 0.01 ^(a)^	0.14 ± 0.01 ^(b)^	0.15 ± 0.01 ^(b)^
GPx [nmol NADPH/min/mg protein]	132.5 ± 16.0	136.9 ± 10.5	182.8 ± 17.4 ^(a)^	162 ± 24.1	162.1 ± 15.5
GR [nmol NADPH/min/mg protein]	56.9 ± 5.9	56.5 ± 5.0	63.3 ± 5.0 ^(a)^	54.9 ± 3.1 ^(b)^	50.5 ± 3.5 ^(b)^
PON1 [μmol phenol/min/mg protein]	138.1 ± 10.3	137.4 ± 8.0	117.6 ± 8.2 ^(a)^	143.4 ± 13.7 ^(b)^	139.9 ± 12.7 ^(b)^
GSH [μmol/g tissue]	3.08 ± 0.16	3.39 ± 0.33	5.55 ± 0.50 ^(a)^	5.42 ± 0.51	4.63 ± 0.32 ^(b)^
DNA damage [arbitrary points]	91.0 ± 4.0	90.9 ± 5.3	174.0 ± 14.4 ^(a)^	183.5 ± 3.0	180.3 ± 2.1

^(a)^—*p* < 0.05 vs. Control. ^(b)^—*p* < 0.05 vs. ROT.

**Table 5:** Effect of cranberry juice (CJ) on oxidative stress markers in the serum of rotenone (ROT) injected rats.

Parameters	Treatment				
	Control	CJ II	ROT	CJ I + ROT	CJ II + ROT
SOD [U/mL]	96.5 ± 10.3	107.1 ± 17.1	85.3 ± 11.2	74.2 ± 11.6	70.9 ± 10.7 ^(b)^
GPx [nmol NADPH/min/mL]	2045.2 ± 143.1	2089.4 ± 92.0	2053.3 ± 254.9	1331.4 ± 70.7 ^(b)^	175.9 ± 38.1 ^(b)^
PON1 [μmol phenol/min/mL]	31.9 ± 4.1	37.7 ± 1.7	34.9 ± 5.5	41.9 ± 5.9 ^(b)^	43.0 ± 5.3 ^(b)^
TAS# [μmol vit C/min/mL]	2.37 ± 0.03	2.30 ± 0.06	1.74 ± 0.09 ^(a)^	1.90 ± 0.04	2.00 ± 0.01 ^(b)^
DNA damage * [arbitrary points]	63 ± 3	72 ± 4	112 ± 4 ^(a)^	119 ± 5	120 ± 4

^(a)^—*p* < 0.05 vs. Control. ^(b)^—*p* < 0.05 vs. ROT #TAS expressed as mmol Trolox/g protein; * DNA damage in leukocytes.

## Data Availability

Original data are available with the authors according to their contribution but not archived in databases elsewhere.
